# Src Family Kinases Regulate Interferon Regulatory Factor 1 K63 Ubiquitination following Activation by TLR7/8 Vaccine Adjuvant in Human Monocytes and B Cells

**DOI:** 10.3389/fimmu.2018.00330

**Published:** 2018-03-01

**Authors:** Lorenza Tulli, Francesca Cattaneo, Juliette Vinot, Cosima T. Baldari, Ugo D’Oro

**Affiliations:** ^1^GSK Vaccines, Siena, Italy; ^2^Department of Life Sciences, University of Siena, Siena, Italy

**Keywords:** toll-like receptor, innate immunity, interferon regulatory factor 1, ubiquitination, TNF receptor associated factor 6, Src family kinases

## Abstract

Toll-like receptors (TLRs) play a key role in the activation of innate immune cells, in which their engagement leads to production of cytokines and co-stimulatory molecules. TLRs signaling requires recruitment of toll/IL-1R (TIR) domain-containing adaptors, such as MyD88 and/or TRIF, and leads to activation of several transcription factors, such as NF-κB, the AP1 complex, and various members of the interferon regulatory factor (IRF) family, which in turn results in triggering of several cellular functions associated with these receptors. A role for Src family kinases (SFKs) in this signaling pathway has also been established. Our work and that of others have shown that this type of kinases is activated following engagement of several TLRs, and that this event is essential for the initiation of specific downstream cellular response. In particular, we have previously demonstrated that activation of SFKs is required for balanced production of pro-inflammatory cytokines by monocyte-derived dendritic cells after stimulation with R848, an agonist of human TLRs 7/8. We also showed that TLR7/8 triggering leads to an increase in interferon regulatory factor 1 (IRF-1) protein levels and that this effect is abolished by inhibition of SFKs, suggesting a critical role of these kinases in IRF-1 regulation. In this study, we first confirmed the key role of SFKs in TLR7/8 signaling for cytokine production and accumulation of IRF-1 protein in monocytes and in B lymphocytes, two other type of antigen-presenting cells. Then, we demonstrate that TLR7 triggering leads to an increase of K63-linked ubiquitination of IRF-1, which is prevented by SFKs inhibition, suggesting a key role of these kinases in posttranslational regulation of IRF-1 in the immune cells. In order to understand the mechanism that links SFKs activation to IRF-1 K63-linked ubiquitination, we examined SFKs and IRF-1 possible interactors and proved that activation of SFKs is necessary for their interaction with TNFR-associated factor 6 (TRAF6) and promotes the recruitment of both cIAP2 and IRF-1 by TRAF6. Collectively, our data demonstrate that TLR7/8 engagement leads to the formation of a complex that allows the interaction of cIAP2 and IRF-1 resulting in IRF-1 K63-linked ubiquitination, and that active SFKs are required for this process.

## Introduction

The innate immune system represents the first defense mecha-nism engaged by the host organism against pathogen infections and is also required to activate adaptive immune responses. A central role in coupling innate and adaptive immunity is played by toll-like receptors (TLRs) (1–4). Following recognition of highly conserved pathogen-associated molecular patterns (5, 6), TLRs activate a signaling cascade that in turn leads to the production of pro-inflammatory cytokines and co-stimulatory molecules, which are required for the response to pathogens (4, 7). Due to their critical role in the activation of the innate immune response TLRs are preferential targets of new vaccine adjuvants based on small molecules (8).

TLR7 and TLR8 belong to a subfamily of endosomal receptors that recognize single-stranded RNAs from viruses as well as endogenous nucleic acids released in the context of patho-genic events (9, 10). TLR7 has been shown to be a good target for a recently described vaccine adjuvant (11, 12). Engagement of these two TLRs by appropriate agonists triggers an intracellular signaling pathway that involves recruitment of the adaptor protein Myeloid differentiation primary response 88 (MyD88), which in turn binds interleukin 1 (IL-1) receptor-associated ki-nase family of protein kinases. Activation of these serine/threonine kinases leads, through TNFR-associated factor 6 (TRAF6), to activation of MAP kinases and nuclear translocation of the transcription factor NF-κB, which are required for induction of inflammatory cytokines. Members of the interferon regulatory factor (IRF) family of transcription factors are also induced by intracellular TLRs such as TLR7 and TLR8, resulting in triggering of other cellular functions associated with these receptors. TLR7 stimulation in plasmacytoid DC activates IRF-7 that in turn leads to IFNα production (13), while in conventional DC, TLR7 engagement is associated to interferon regulatory factor (IRF-1) induction, which in these cells controls cytokine gene expression (14–16). Src family kinases (SFKs) were also shown to participate in TLRs signaling (17–24). Activity of these kinases is finely regulated by a balance of phosphorylation and dephosphorylation events (25). Phosphorylation of their carboxyl-terminal tyrosine residue (Tyr-530 in human c-Src), mainly by C-terminal Src kinase, leads to an intramolecular interaction of this phosphorylated residue with the Src homology (SH) 2 domain, resulting in an enzymatically inactive closed conformation of SFKs. Dephosphorylation of the carboxyl-terminal tyrosine residue by several phosphatases or displacement of the intramolecular SH2 domain by another protein with a higher affinity SH2 do-main opens up the kinase domain, which becomes catalytically active. This event allows intermolecular autophosphorylation of a tyrosine residue in the activation loop (Tyr-419 in human c-Src), which locks the catalytic pocket into an open conformation and is required for maximum activation of these kinases. PP2 is a small molecule inhibitor of SFKs, which binds specifically these kinases, preventing the auto-phosphorylation in the activation loop that is necessary for the full activation of SFKs (26). We previously demonstrated that pharmacological inhibition of SFKs with PP2 in human monocyte-derived dendritic cells (MoDCs) impaired TLR7/8-mediated release of several pro-inflammatory cytokines by interfering with the accumulation of IRF-1, thus suggesting an involvement of SFKs in IRF-1 regulation and TLR7/8 signaling (16).

Interferon regulatory factor 1 is the prototype member of a family of nine transcription factors (27, 28) and is expressed in a variety of cells in which plays a crucial role in promoting the expression of type I IFN genes following viral infection (29–31). Similar to other transcription factors, IRF-1 is tightly regulated at both transcriptional and posttranslational levels (32–35). In particular, IRF-1 can undergo K48-ubiquitination to be degraded *via* the proteasome pathway (36), or K63-ubiquitination through the recruitment of TRAF6 and cIAP2 to become activated following IL-1R stimulation (37).

Here, we show that a similar signaling pathway involving TLRs and SFKs controls IRF-1 expression and cytokine production in two other important classes of antigen-presenting cells, namely monocytes and B-lymphocytes, which are key target for vaccine adjuvants. Moreover, we provide evidence that SFKs control the TLR7/8-dependent release of pro-inflammatory cytokines by monocytes and B-lymphocytes by promoting K63-linked ubiquitination of IRF-1. Finally, we demonstrate a crucial role of SFKs in binding and activating the ubiquitin ligase TRAF6 and that its inhibition impairs the formation of a com-plex with cIAP2 and IRF-1 that is a crucial step for IRF-1 K63-linked ubiquitination.

## Materials and Methods

### Cell Cultures

Human embryonic kidney cells stably expressing human TLR7 (hTLR7-HEK293 cells) were cultured in DMEM containing 4.5 g/ml glucose, supplemented with 10% heat inactivated FBS, 100 U/ml penicillin, 100 µg/ml streptomycin, 2 mM glutamine, 5 µg/ml puromycin, 5 µg/ml blasticidin, and 2 mM glutamine. Human monocytic leukemia cell line THP-1 were cultured in RPMI 1640 containing 2.5 g/l glucose, supplemented with 10% heat inactivated FBS, 10 mM HEPES, 10 mM Sodium Pyruvate, 100 U/ml penicillin, and 100 µg/ml streptomycin.

PBMCs were isolated from buffy coats of healthy donors using Ficoll gradient. Human primary B cells were purified by negative selection using the RosetteSep B-cell enrichment Cocktail (StemCell Technologies) followed by density gradient centrifugation on Lympholite (Cedarlane Laboratories) as previously described (38). Monocytes were isolated from purified human PBMC using anti-CD14 magnetic beads (Miltenyi Biotec). Informed consent was obtained according to the Declaration of Helsinki.

Epstein–Barr virus (EBV)-immortalized B cell line and human primary B cells were cultured in RPMI 1640 (Sigma-Aldrich, The Woodlands, TX, USA) supplemented with 7.5% bovine calf serum at 37°C in a humidified atmosphere with 5% CO2.

### Cell Treatment and Cytokine Detection

Cells were treated with 20 µM PP2 (Sigma-Aldrich) or DMSO for 30 min then stimulated with R848 (10 µM) (Invivogen) overnight. Supernatants were collected and the amount of inflammatory cytokines was measured using Mesoscale Assay Human-Pro-inflammatory 7-spot (Meso Scale Discovery), following the manufacturer’s instructions.

### Cell Transfection

hTLR7-HEK293 cells were seeded in 100 mm diameter culture dishes (3 × 10^6^ cells/dish) and after 24 h were transfected using Lipofectamine 2000 (Invitrogen) with an expression plasmid encoding hemagglutinin (HA)-tagged K48-only or K63-only ubiquitin (kindly provided by Dr. Jonathan Ashwell, NCI, NIH, Bethesda, MD, USA). Four days post-transfection cells were treated as described above. THP-1 cells were seeded in 100 mm diameter culture dishes (6 × 10^6^ cells/dish) and immediately transfected by Lipofectamine 2000 (Invitrogen) with an expression plasmid encoding HA-tagged Ubiquitin. After 24 h, cells were treated as described above.

### Immunoprecipitation and Immunoblot Analysis

Cells were treated with 20 µM PP2 at concentration or DMSO for 30 min prior to incubation with R848 (10 µM) for 2 h, then lysed with a buffer containing 150 mM NaCl, 20 mM Tris–HCl, Triton X-100 1%, 1 µg/ml pepstatin A, 1 µg/ml leupeptin, 1 µg/ml aproteinin, 200 µg/ml sodium orthovanadate, 1 mM phenylmethylsulfonyl fluoride, and 1 mg/ml of *N*-ethylmaleimide, for 5 min on ice. Lysates were centrifuged for 20 min at 21,000 × *g* at 4°C. Protein concentration was measured using the Bradford assay (Sigma-Aldrich). Proteins were separated by 10% SDS-PAGE electrophoresis using the NuPage Gel System, according to the manufacturer’s instructions and transferred to nitrocellulose membranes for Western blot analysis. After blocking with PBS containing 0.05% Tween 20 (PBST) and 5% BSA (Sigma-Aldrich), proteins were detected with specific antibodies.

For immunoblot and immunoprecipitation analysis, we used the following antibodies: anti-IRF-1 (cat# 8478 Cell Signaling for immunoblot and cat# sc-497 Santa Cruz Biotechnology for immunoprecipitation); anti-TRAF6 (cat# 8028 Cell Signaling); anti-Src (cat# 2108 Cell Signaling); anti-p-SFKs (Tyr416) (2101 Cell Signaling); anti-p-Tyrosine (cat# 8954 Cell Signaling); anti-HA (cat# 2367 Cell Signaling); anti-cIAP2 (cat# ab32059 Abcam), anti-β-actin (cat# ab8227 Abcam); and HRP-conjugated secondary antibodies (DAKO).

The same filters were stripped and re-probed with an anti-actin antibody (cat#mab1501 Millipore) for loading control. Immunoblots were captured for densitometry using ImageJ software, and each signal was normalized to its respective protein signal on the same blot.

For immunoprecipitations, cell lysates were incubated overnight at 4°C with the primary antibody crosslinked with the BS3 reagent to Dynabeads^®^ Protein G Immunoprecipitation Kit (Life Technologies). Crosslinking avoid the co-elution of IgGs with the targed antigen. Immunoprecipitated complexes were eluted and analyzed by Western blot. To analyze TRAF6 tyrosine phosphorylation, lysates were immunoprecipitated with anti-TRAF6 antibody. The immune complexes immobilized on Dynabeads were then resuspended in 2% SDS and heat-treated (90°C for 5 min) to disrupt the interaction between TRAF6 and SFKs. The protein mixture was diluted in PBS to reduce the SDS to a final concentration of 0.01%, and re-immunoprecipitated with the same anti-TRAF6 antibodies. The re-immunoprecipitated samples were eluted and analyzed by western blot. To control for efficiency of immunoprecipitation and protein loading and transfer, the blots were stripped and re-probed with the antibody used for immunoprecipitation. Immunoblots were captured for quantitative densitometry using ImageJ software, and each signal was normalized to its respective protein signal on the same blot.

### RNA Purification and Quantitative RT-PCR

Total RNA was extracted from THP-1, human B cells and HEK-293 using the RNeasy Plus Mini Kit (Qiagen) and retrotranscribed using the iScript™ cDNA Synthesis Kit (Bio-Rad, Hercules, CA, USA). Three independent reverse transcription reactions were performed on each sample. Real-time quantitative PCR (qRT-PCR) was performed in triplicate on each cDNA in 96-well optical PCR plates (Sarstedt, Nümbrecht, Germany) using SSo Fast EvaGreenR SuperMix (Bio-Rad) and a CFX96 Real-Time system (Bio-Rad). Results were processed and analyzed using CFX Manager Version 1.5 software (Bio-Rad). Transcript levels were normalized to HPRT1 used as housekeeping control for all the reactions. The primers sequences are as follow: HPRT1 forward 5’-AGATGGTCAAGGTCGAAG-3’ and reverse 5’-GTATTCATTATAGTCAAGGGCATAT-3’; IRF-1 for-ward 5’-CGTGGGACATCAACAAGGA-3’ and reverse 5’-GTGGAAGCATCCGGTACACT-3’.

### Statistical Analysis

Mean values, SDs and Student’s *t-*test (unpaired) were calculated using Microsoft Excel Version 14.0.0 software (Microsoft) (Redmont, WA, USA). A *P* < 0.05 was considered statistically significant.

## Results

### Activation of SFKs Is Required for Inflammatory Cytokine Production in both Monocyte-Like Cells and Primary B Cells

We have previously demonstrated that SFKs activity is required for the release of pro-inflammatory cytokines by human Mo-DCs stimulated with the imidazoquinoline compound R848 (Resiquimod), an agonist for human TLR7 and TLR8 (16). To un-derstand whether this mechanism is shared by other immune cells, which are also targets for a TLR7/8 agonist vaccine adjuvant, we extended our analysis to monocytes and B-lymphocytes.

**Figure 1:**
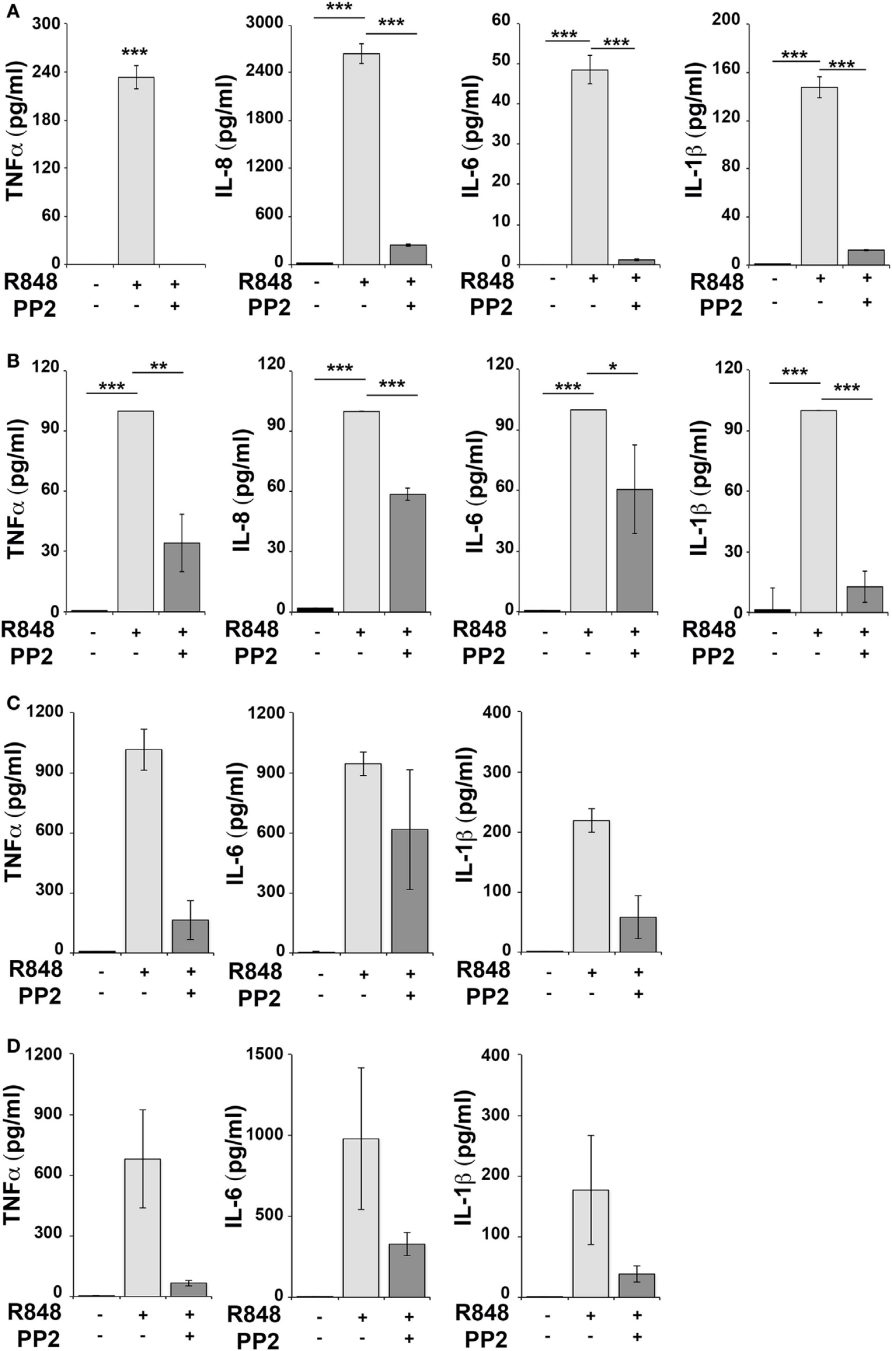
Src family kinases inhibition results in decrease of pro-inflammatory cytokine production in response to TLR7/8 stimulation in monocytes and B cells. THP-1 **(A)** and primary human B cells **(B)** were pretreated with PP2 (20 µM) or DMSO alone for 30 min and stimulated with R848 (10 µM) for 16 h. Monocytes **(C,D)** were pretreated with PP2 (20 µM) or DMSO alone for 30 min and stimulated with R848 (1 µM) for 16 h. Cytokine levels in the supernatants were quantified by Meso Scale Discovery immunoassay. Cells treated with vehicle only (DMSO) were used as control (Ctr). **(A,B)** Values represent the mean ± SD of cytokine concentration (picogram per milliliter) in the culture supernatants (*n* ≥ 3), ***P < 0.001, **P < 0.01, *P < 0.05. Results are representative at least of three independent experiments. **(C,B)** The histograms show the mean (from duplicate samples) ± SD of cytokine concentration (pg/ml) in the culture supernatants from two different donors respectively in panels **(C,D)** and are representative of four experiments performed on monocytes from four different donors.

Human monocytic THP-1 cells and human primary monocytes and B cells were stimulated for 16 h with R848 in the absence or in the presence of the SFKs-specific inhibitor PP2 and cell supernatants were collected and analyzed using the multiplex Meso Scale Discovery immunoassay to measure the levels of 7 inflammatory cytokines in the culture supernatants. As shown in Figure 1, we observed a strong induction in the release of 4 of the 7 pro-inflammatory cytokines analyzed, specifically TNFα, IL-6, IL-8, and IL-1β in THP-1 cells (panel A) and in primary B cell (panel B), while in response to R848 only TNFα, IL-6 and IL-1β were released by human primary monocytes (panel C). However, in all cases, cytokine production was largely reversed when SFKs were inhibited by PP2 pretreatment.

Hence, SFKs activation is a critical event for cytokine production also in human monocytes and B cells.

### SFKs Play a Key Role in the Accumulation of IRF-1 in THP-1 and B Cells

Analysis of the signaling pathways triggered in MoDCs by R848 identified IRF-1 as the main effector responsible for the transcription of pro-inflammatory genes. Activation of this transcription factor was directly correlated with the activation of SFKs (16).

**Figure 2:**
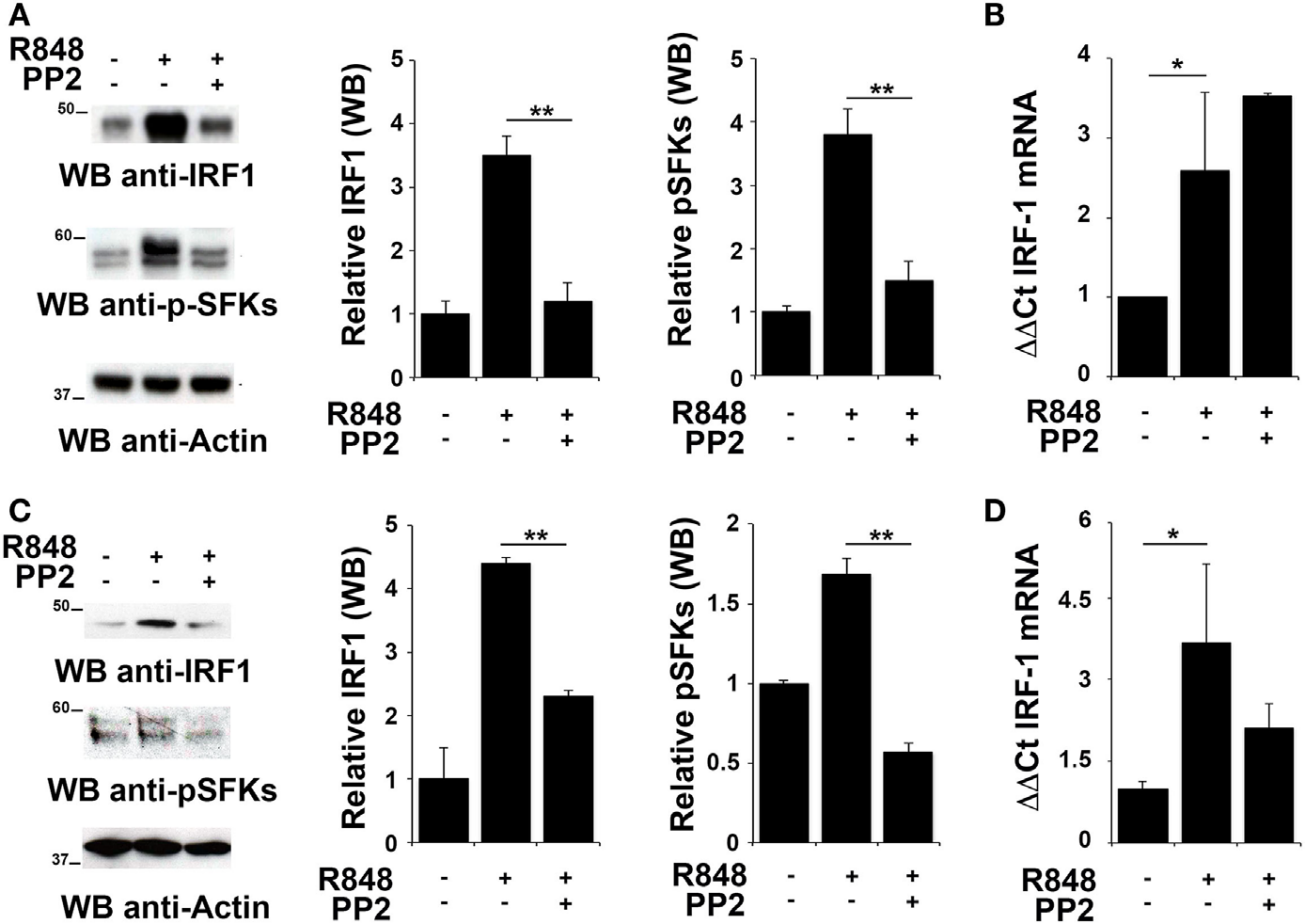
Src family kinases are required for interferon regulatory factor 1 (IRF-1) protein accumulation in response to TLR7/8 stimulation in monocytes and B cells. **(A,C)** Immunoblot analysis of IRF-1 and p-SFKs in THP-1 **(A)** and primary B cells **(C)** pretreated with PP2 (20 µM) or DMSO alone and stimulated with R848 (10 µM) for 2 h. After stripping, filters were re-probed with an antibody against actin as loading control. Results are representative at least of three independent experiments. The histograms on the right of the blot show the results of the densitometric analysis on three independent experiments. Each signal was normalized to its respective actin signal on the same blot and expressed as fold induction (mean ± SD) compared to untreated sample. **(B,D)** qRT-PCR analysis of *lRF-1* mRNA in THP-1 **(B)** and human B cells **(D)** pretreated with PP2 (20 µM) or DMSO alone and stimulated with R848 (10 µM) for 2 h. The relative abundance of the gene transcripts was determined on triplicate samples from at least three independent experiments using the ΔΔCt method and is expressed as the normalized fold expression (mean ± SD) compared to untreated sample. **P < 0.01, *P < 0.05.

To evaluate whether IRF-1 was similarly involved in the re-lease of pro-inflammatory cytokines in THP-1 and B cells, we analyzed its expression and activation in these cells after R848 stimulation. As shown in Figures 2A,C, stimulation of THP-1 cells or primary B cells with R848 for 2 h resulted in IRF-1 accumula-tion (upper western blot and left histogram in Figures 2A,C), which directly correlated with an increase in the auto-phosphorylation of SFKs on the activator Tyr residue (Y419 in c-Src) (middle western blot and right histogram in Figures 2A,C), that indicates activation of this type of kinases. These effects were all inhibited by pretreatment with PP2, confirming the importance of SFKs activation in the up-regulation of this transcription factor.

We next measured IRF-1 mRNA levels by qRT-PCR in cells treated as described above to assess whether the changes in IRF-1 expression were dependent on transcriptional regulation of the respective gene by SFKs. The level of *IRF1* mRNA was increased following stimulation with R848 in THP1 cells (Figure 2B) and primary B cells (Figure 2D). Interestingly, no decrease in the transcript was observed in either cell types when cells were pre-treated with PP2, suggesting that, as previously shown for MoDCs, in these cells SFKs regulate IRF-1 expression posttranscriptionally The analysis was also extended to EBV-immortalized primary human B lymphocytes (EBV-B). Interestingly, no changes in the mRNA levels were detected in these cells treated with R848, either in the presence or absence of PP2 (Figure S1A in Supplementary Material). Nevertheless, similar to THP-1 and primary B cells, we observed an accumulation of IRF-1 protein in R848-treated EBV-B cells that was blocked by PP2 (Figure S1B in Supplementary Material).

Collectively, these results support the hypothesis that posttranslational modifications, in addition to transcription activation, lead to IRF-1 accumulation as a consequence of SFKs activation.

### SFKs Activation Is Essential for K63-Linked Ubiquitination of IRF-1

To elucidate the mechanism responsible for IRF-1 downregulation in PP2 treated cells, we asked whether SFKs might prevent IRF-1 degradation by promoting posttranslational modifications in the protein. In fact, it has been reported that IRF-1 can undergo several posttranslational modifications, including sumoylation and ubiquitination (35). K48-linked ubiquitination of IRF-1 has been shown to target the protein to the proteasome for degradation (36) whereas K63-linked ubiquitination is essential for IRF-1 activity (37).

**Figure 3:**
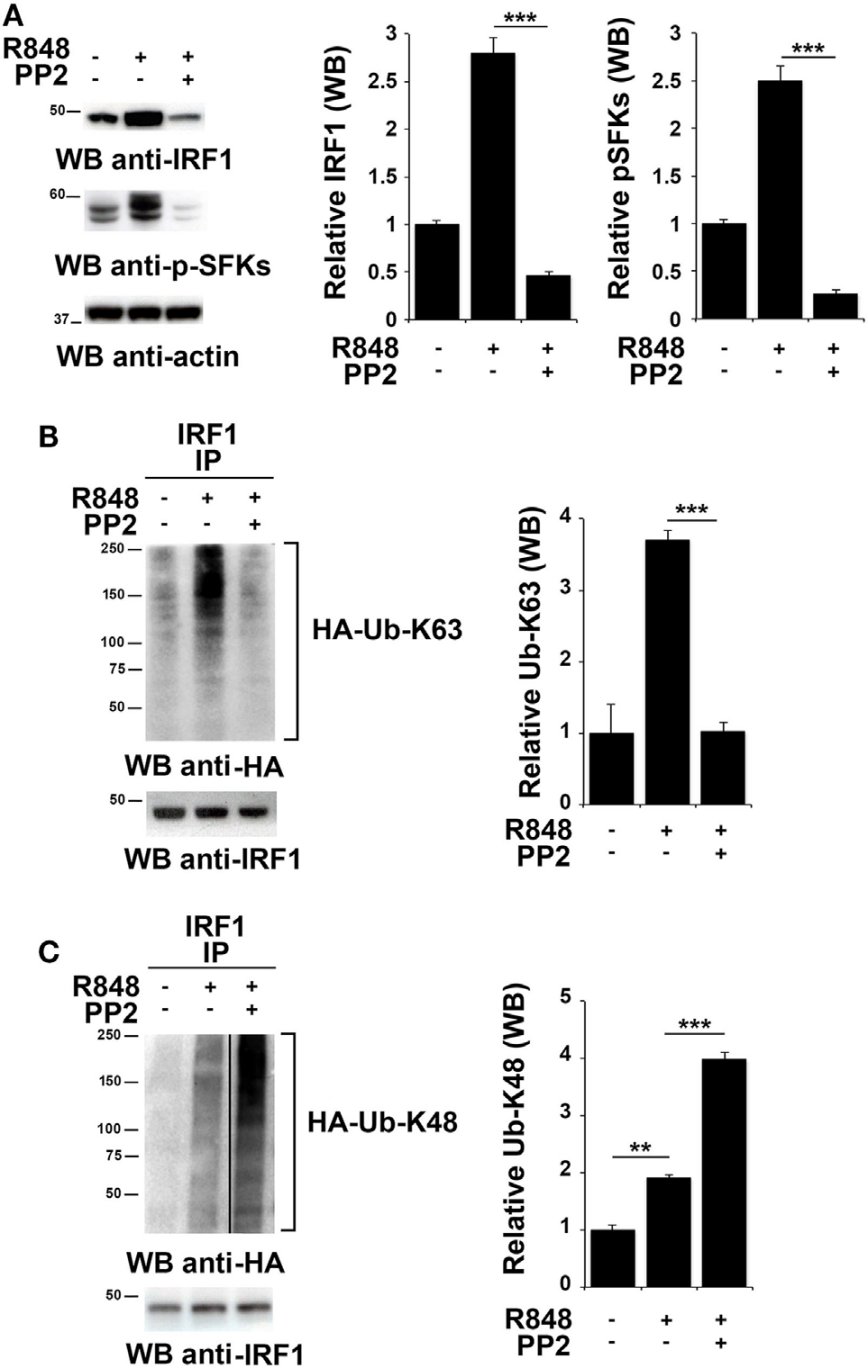
Src family kinases activation is essential for interferon regulatory factor 1 (IRF-1) K63-linked ubiquitination. **(A)** Immunoblot analysis of IRF-1 and pSFKs in hTLR7-HEK293 cells pretreated or not with PP2 (20 µM) for 30 min and stimulated with R848 (10 µM) for 2 h. After stripping, membranes were blotted with anti-actin antibody as loading control. The histograms on the right of the blots show the results of the densitometric analysis on three independent experiments. Each signal was normalized to its respective actin signal on the same blot and expressed as fold induction (mean ± SD) compared to untreated sample. **(B,C)** hTLR7-HEK293 cells transiently transfected with plasmids coding for hemagglutinin (HA)-tagged K63-only **(B)** or K48-only ubiquitin mutants **(C)** were pretreated or not with PP2 (20 µM) for 30 min and stimulated for 2 h with R848 (10 µM). Total cell lysates were immunoprecipitated using an anti-IRF-1 antibody, separated by SDS-PAGE, and immunoblotted using anti-HA antibody for ubiquitin detection. Membranes were stripped and re-probed with an anti-IRF-1 antibody as control (bottom panels). The histograms on the right of the blots show the results of the densitometric analysis on three independent experiments. Each signal was normalized to the signal of the protein used for the pull-down and expressed as fold induction (mean ± SD) compared to untreated sample. ***P < 0.001, **P < 0.01, *P < 0.05.

To investigate whether SFKs modulate IRF-1 ubiquitination upon TLR7/8 engagement, we used HEK293 cells stably expressing human TLR7 (hTLR7-HEK293) as a specific cell model for studying the TLR7 signaling cascade. As observed in immune cells, stimulation of these cells with R848 led to an accumulation of IRF-1 that was reversed by PP2 treatment that also resulted in the inhibition of SFKs phosphorylation (Figure 3A). To analyze IRF-1 ubitiquination, hTLR7-HEK293 cells were transiently transfected with plasmids coding for HA-tagged K63-only or HA-tagged K48-only ubiquitin mutants and cell lysates were subjected to immunoprecipitation using an anti-IRF-1 antibody. As shown in Figure 3B, R848 stimulation resulted in an increase in the level of K63-linked ubiquitination of IRF-1, which was drastically reduced by pretreatment with PP2. Conversely, K48-linked ubiquitinated IRF-1 accumulated when cells were preincubated with PP2 (Figure 3C). It should be noted that, to normalize the levels of immunoprecipitated IRF-1 and thus avoiding that differences in the levels of ubiquitination could depend on the different levels of IRF-1 in the immunoprecipitates, a limiting amount of antibody was used to perform the immunoprecipitation. This resulted in similar levels of immunoprecipitated IRF-1 in all samples (lower blots in Figures 3B,C). Similar results were observed in THP-1 cells transiently transfected with HA-tagged K63-only or K48-only ubiquitin mutants (Figure S2 in Supplementary Material). To confirm the specificity of these results, we immunoprecipitated the lysates of hTLR7-HEK293 cells transiently transfected with HA-tagged K63-only or HA-tagged K48-only ubiquitin mutants with an anti-HA antibody and then we processed the immunoprecipitates for IRF-1 immunoblotting (Figure S3 in Supplementary Material). Also in this case TLR7 engagement increased the levels of IRF-1 in the K63 ubiquitinated pool, which were inhibited by PP2 treatment, while Src kinase inhibition resulted in an expansion of the K48 ubiquitinated form. Taken together, these findings indicate that SFKs play a crucial role in the regulation of the balance between accumulation and degradation of IRF-1.

### SFKs Activation Regulates the Interaction of IRF-1 with cIAP2 and TRAF6

**Figure 4:**
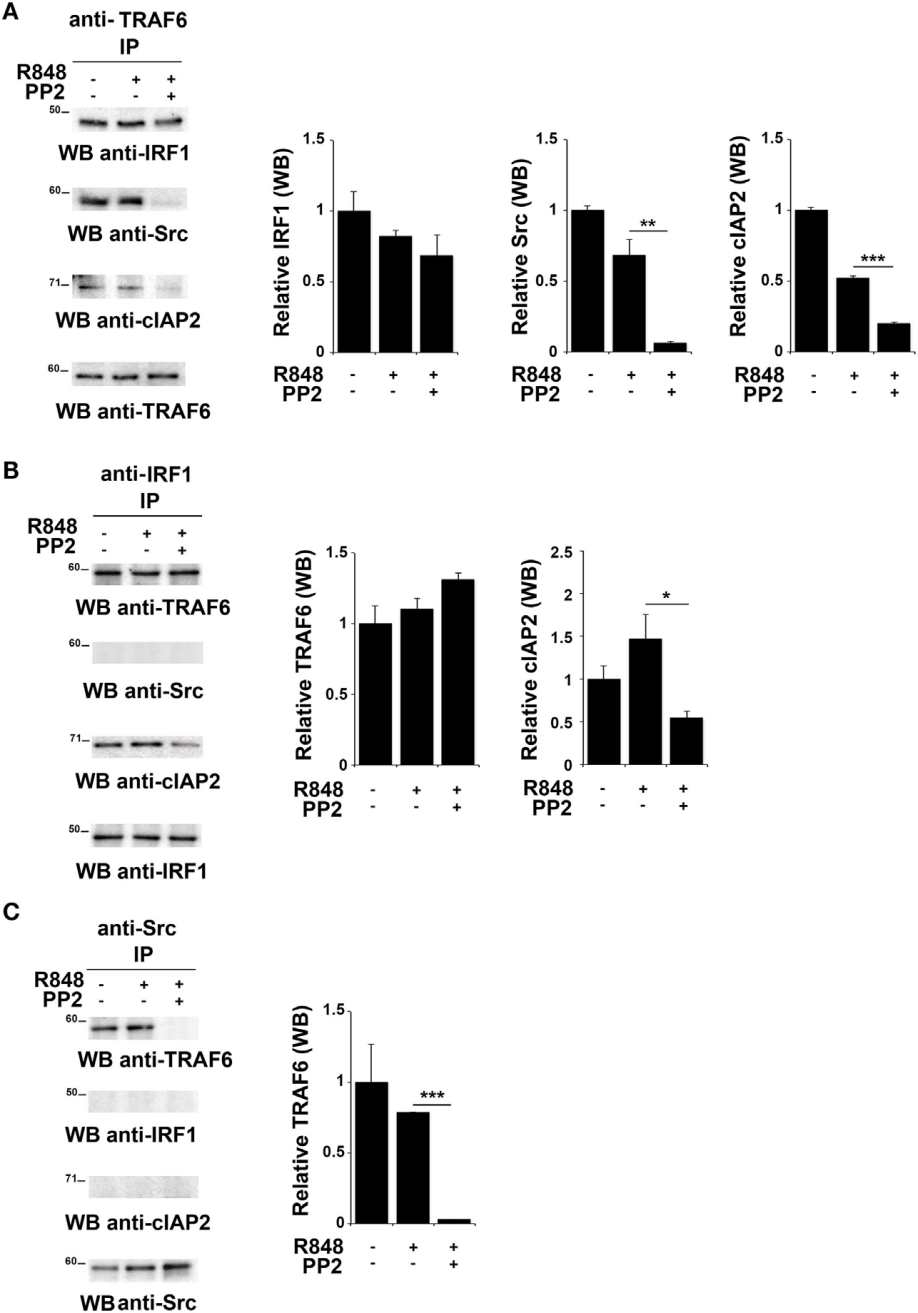
Inhibition of SFKs impairs the stability of the SFKs-TRAF6-IRF-1-cIAP2 complex **(A–C)** hTLR7-HEK293 cells were pretreated or not with PP2 (20 µM) for 30 min and stimulated for 2 h with R848 (10 µM). Total cell lysates were immunoprecipitated using an anti-TRAF6 **(A)**, anti-interferon regulatory factor 1 (IRF-1) **(B)**, or anti-pSFKs **(C)** antibody, respectively. Co-immunoprecipitated cIAP2, TNFR-associated factor 6 (TRAF6), SFKs, and IRF-1 proteins were detected by immunoblot. Histograms on the right side of the blots show the quantification of the relative amount of the pulled-down proteins, calculated on three independent experiments. Each signal was normalized to the signal of the protein used for the pull-down and expressed as fold induction (mean ± SD) compared to untreated sample. ***P < 0.001, **P < 0.01, *P < 0.05.

TNFR-associated factor 6 is an ubiquitin ligase that catalyzes the K63-linked ubiquitination of several signaling molecules (39–42). Of note, it has been previously reported that TRAF6 recruits the E3 ligase cIAP2 and IRF-1 in response to IL-1, forming a complex that allows K63-linked ubiquitination of IRF-1 by cIAP2 (37). Moreover, Liu A. et al. have demonstrated a functional association of SFKs and TRAF6 during TLR4 signaling (43). To establish whether TRAF6 participates in SFKs-dependent IRF-1 regulation following TLR7/8 activation, lysates from hTLR7-HEK293 cells stimulated with R848 in the presence or absence of PP2 were subjected to co-immunoprecipitation experiments to assess protein–protein interactions. Analysis of TRAF6-specific immunoprecipitates showed that this protein forms a complex with IRF-1, Src, and cIAP2 at steady state as well as following R848 stimulation. However, treatment with PP2 impaired the binding of TRAF6 with Src and cIAP2 (Figure 4A), thus showing that activation of SFKs is required for a stable interaction within this complex. On the other hand, when IRF-1 immunoprecipitates were evaluated, only TRAF6 and cIAP2 were found in the complex, while no Src could be detected either before or after TLR7 triggering (Figure 4B). The absence of Src in this complex could depend on the fact that the kinase directly interacts with TRAF6 and that this interaction is not preserved in the anti-IRF-1 immunocomplex. Nevertheless, inhi-bition of SFKs by PP2 reduced the amount of associated cIAP2. Western blot of Src co-immunoprecipitated molecules confirmed that the kinase activity is required for TRAF6 binding (Figure 4C). Collectively, these results provide evidence that under basal conditions TRAF6 is associated with IRF-1, cIAP2, and SFKs in a SFKs activity-dependent manner.

### TLR7 Engagement Leads to Src K63-Linked Ubiquitination and TRAF6 Tyrosine Phosphorylation

**Figure 5:**
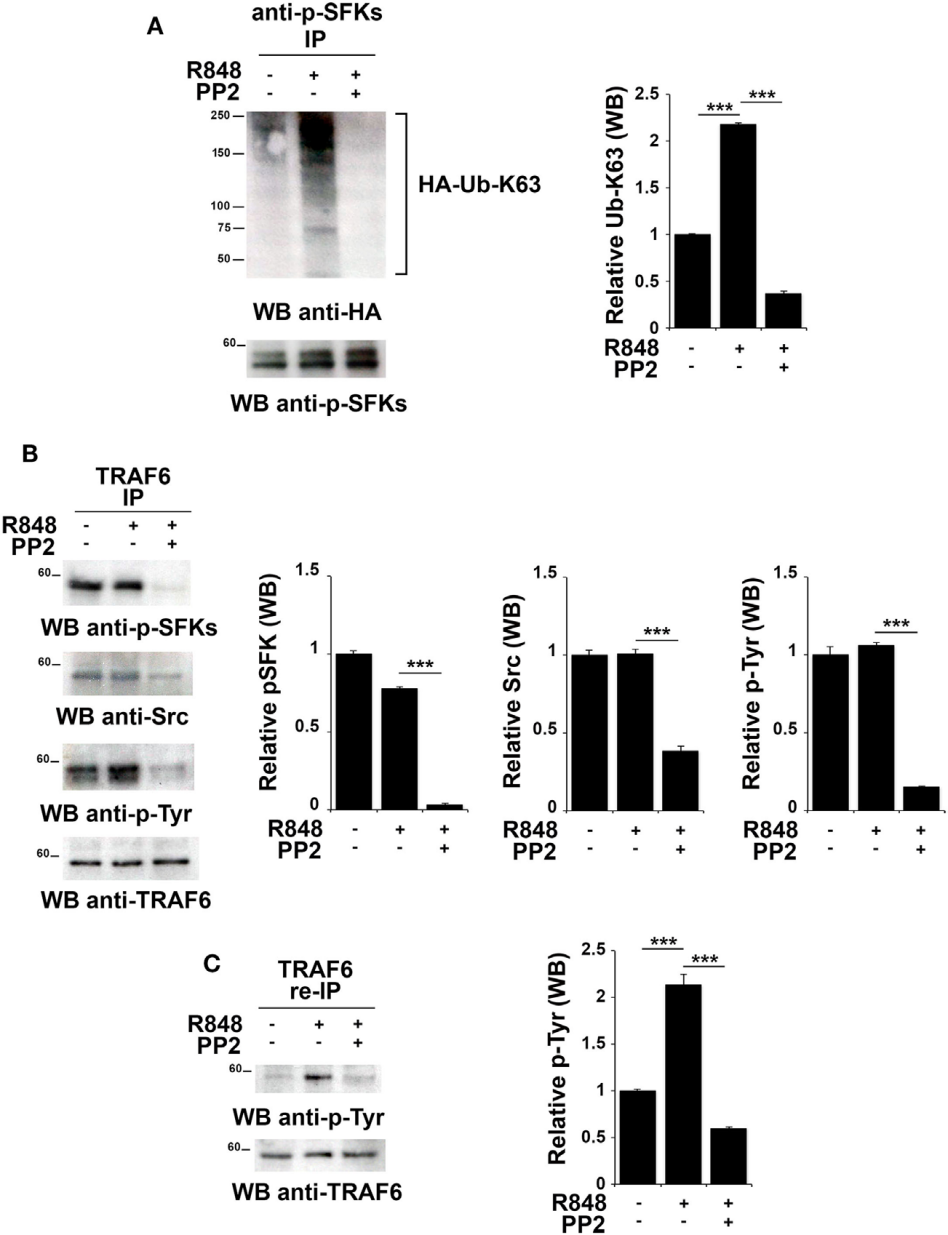
TLR7 triggering leads to tyrosine kinase dependent Src K63-linked ubiquitination and TNFR-associated factor 6 (TRAF6) tyrosine phosphorylation. hTLR7-HEK293 cells, transiently transfected with plasmids coding for hemagglutinin (HA)-tagged K63-only, were pretreated or not with PP2 (20 µM) for 30 min and stimulated for 2 h with R848 (10 µM). **(A)** Total cell lysates were immunoprecipitated with anti-pSFKs antibody and immunoblotted using anti-HA antibody for ubiquitin detection. After stripping, the presence of p-SFKs was assessed by immunoblot analysis using specific antibodies, as control. **(B)** Total cell lysates were immunoprecipitated with anti-TRAF6 and immunoblotted with antibodies against pSFKs, Src, and pTyr. **(C)** TRAF6-specific immunoprecipitates were eluted with 2% SDS in order to disrupt the TRAF6-SFKs interaction and after dilution with PBS re-immunoprecipitated with anti-TRAF6 antibody. Results are representative of three independent experiments. Histograms on the right side of the blots show the quantification of the pulled-down proteins, calculated at least on two independent experiments. Each signal was normalized to the signal of the protein used for the pull-down and expressed as fold induction (mean ± SD) compared to untreated sample. ***P < 0.001, **P < 0.01.

Src family kinases have been proposed to promote TRAF6 phosphorylation that in turn can modify SFKs by adding K63-linked ubiquitin chains (18, 43). Therefore, we tested whether this mechanism might also occur following TLR7/8 activation. Total lysates from hTLR7-HEK293 cells transiently transfected with plasmids coding for HA-tagged K63-only were subjected to immunoprecipitation experiments using an anti-pSFKs antibody and then probed by immunoblot with an anti-HA antibody. As shown in Figure 5A, we found that R848 stimulation increases the level of Src K63-linked ubiquitination, a modification which is completely abrogated by inhibition of kinase activity by PP2.

To understand whether in turn, TRAF6 might be regulated by SFKs, we immunoprecipitated TRAF6 from hTLR7-HEK293 cells stimulated with R848 and pretreated or not with PP2. Immunoblot analysis with an anti-phospho-tyrosine antibody showed that TRAF6 was phosphorylated upon TLR7/8 stimulation in a kinase activity-dependent manner. Blotting this immunoprecipitate with an antibody against the activator Tyr residue (Y419) of Src confirmed that the active kinase is bound to TRAF6 in a kinase activity-dependent manner (Figure 5B). To confirm that the tyrosine phosphoprotein detected in TRAF6-specific immunoprecipitates is indeed TRAF6 and not the co-immunoprecipitated and co-migrating tyrosine phosphorylated SFKs, the immunoprecipitated samples were treated with 2% SDS to disrupt the TRAF6/pSFKs interaction and re-immunoprecipitated using an anti-TRAF6 antibody to purify TRAF6 from all co-immunoprecipitated proteins. Probing these samples with an anti-phospho-tyrosine antibody clearly proved that TRAF6 is phosphorylated upon stimulation of TLR7/8, and this event is completely blocked by PP2 (Figure 5C) indicating that TRAF6 phosphorylation is mediated by SFKs. Membranes were stripped and re-probed with an anti-pSFKs antibody to verify the absence of pSFKs (data not shown).

Collectively, our results provide evidence that following TLR7/8 engagement the association between SFKs and TRAF6 leads to K63-linked ubiquitination of SFKs, which in turn is responsible for the phosphorylation of a tyrosine residue on TRAF6. We have shown that these modifications are crucial for the formation of a complex between IRF-1, TRAF6 and cIAP2 and lead to IRF-1 K63-linked ubiquitination. This posttranslational modification has a crucial role in IRF-1 stabilization, which is required for the nuclear translocation of the transcription factor and the subsequent activation of its target genes.

## Discussion

In this study, we demonstrate that SFKs play an essential role in K63-linked ubiquitination of IRF-1 in response to TLR7/8 triggering. We had previously reported that activation of SFKs is required for the release of pro-inflammatory cytokines and the accumulation of IRF-1 during TLR7/8 signaling in human MoDCs (16). Here, we first show that this mechanism is also shared by other cells of the immune system, namely monocytes and B-lymphocytes, which are potential target of TLR7/8 agonists vaccine adjuvants. Moreover, we demonstrate that Src kinase associates with TRAF6 in a complex, which also contains IRF-1 and cIAP2, and that following TLR7 triggering Src undergoes K63-linked ubiquitination and phosphorylates TRAF6. These events lead to K63-linked ubiquitination of IRF-1 in the complex, which is responsible for the increased levels of this transcription factor elicited by TLR7 engagement.

Interferon regulatory factors (IRFs) play a crucial role in the connection between innate and adaptive immunity by modulating the activation of immune cells triggered by TLRs (28). Indeed, TLR signaling leads to the transcriptional activation of a variety of genes as a result of the increase in the levels of specific transcription factors (34, 44). To enhance the availability of a particular transcription factor, the cells can adopt two mechanisms: one is to increase the rate of transcription of the gene encoding that particular protein, the other is by promoting its stability and activation. The latter mechanism is mainly achieved by specific posttranslational modifications, which may prevent protein degradation and increase protein half-life, or they may promote or inhibit protein–protein interactions allowing nuclear translocation and binding to the regulatory regions of the target genes. This type of mechanisms plays a critical role in the regulation of IRF-1 (45) in addition to its control at the transcriptional level (46). Under basal conditions, the rate of IRF-1 turnover is high (half-life 20–40 min), such that its steady-state levels are maintained very low. Indeed, this transcription factor contains ubiquitination and degradation signals within its C-terminal region, which lead to its constitutive ubiquitination and degradation by the proteasome pathway (34–36). It is known that the IRF-1 levels rapidly increase in response to stimuli such as viral infection, DNA damage, or TLR stimulation (16, 47, 48) due not only to its transcriptional activation but also to changes in its posttranslational modification that greatly increase the protein stability.

To understand how SFKs modulate IRF-1 accumulation following TLRs engagement, we first measured the mRNA levels of IRF-1 in cells stimulated with the TLR7/8 agonist R848 and pretreated or not with the SKFs inhibitor, PP2. Because no differences in IRF-1 transcript levels were found, we hypothesized that SFKs may be involved in the posttranslational regulation of IRF-1, in particular, in the ubiquitination of this transcription factor. Ubiquitin has a central role in many cellular functions, such as signal transduction, receptor downregulation, protein–protein interaction, protein transport and degradation and gene transcription, which may be regulated by different types of ubiquination leading to different ubiquitin chains (49–51). The best characterized type of ubiquitin chain is formed by several ubiquitins linked through lysine 48 (K48) and is typically associated with proteasome-mediated protein degradation (49, 51). Moreover, several proteins may be K63-linked ubiquitinated. This modification is known to be important in several cellular processes and, in particular, in the signal transduction of various activation pathways (49, 51). The activation and degradation of several IRFs, such as IRF5, IRF7 (39, 52, 53), and also IRF-1 (36, 37), are regulated by both types of ubiquitination. Using hTLR7-HEK293 cells transiently transfected with plasmids encoding ubiquitin mutants that form only K63 or K48-linked chains, we found that activation of SFKs results in an increase in K63-linked ubiquitination of IRF-1, which is reversed by kinase inhibition following PP2 treatment. On the contrary, the levels of K48-linked ubiquitinated IRF-1 were found to be increased by PP2 treatment. These results suggest that, upon TLR7 engagement, IRF-1 preferentially undergoes K63-linked ubiquitination and that this modification rescues it from degradation. Hence, consistent with our current understanding of IRF-1 regulation, the protein levels of IRF-1 are controlled posttranslationally by K48-linked ubiquitination and proteasomal degradation (36). Once SFKs are activated by TLR7/8, they trigger an increase in K63-linked ubiquitination of IRF-1, resulting in the activation and accumulation of the transcription factor. Interestingly, several studies proved that both types of ubiquitination target the same residues in the DNA-binding domain of IRF-1. In particular Narayan et al. (54) suggested the possibility that K63-ubiquitination may counteract the constitutive K48-ubiquitination of IRF-1. We may, therefore, hypothesize that the two events compete, such that cells promote K48- or K63-linked ubiquitination depending on the external conditions.

It has been reported that IL-1-induced K63-linked ubiquitination of IRF-1 is catalyzed by the E3 ligase cIAP2, which in turn is activated through its ubiquitination by TRAF6. Moreover, these three proteins have been shown to interact with each other (43). TRAF6 is one of the major signal transducers of the TNF receptor family (42, 55) and has long been known to participate in TLR signaling, ubiquitinating itself and several proteins, including some IRFs (37, 39, 41). cIAP2, instead, not only regulates caspases and hence apoptosis, but has also been implicated in the modulation of inflammatory signaling (50, 56). We tested whether these proteins are also recruited to K63-linked ubiquitinated IRF-1 during TLR7/8 signaling and the role played by SFKs in regulating these interactions. We found that cIAP2 is able to bind TRAF6 and IRF-1, and that these interactions are affected by PP2, suggesting that the activation of SFKs is an upstream essential step in this signaling cascade. While TRAF6 also interacts with IRF-1, this event was found to be independent of SFKs activation. Finally, stimulation with TLR7/8 agonists leads to reciprocal modification of TRAF6 and SFKs in a SFKs activity-dependent manner. Reciprocal binding and modification between TRAF6 and SFKs have been extensively described for different signaling pathways in multiple cells of the immune system, although the hierarchical position between these two molecules remains still unclear. In particular, it was reported that the interaction between TRAF6 and c-Src requires an active SFK kinase domain and that the polyproline sequence of TRAF6 and the Src-homology 3 (SH3) domain of Src are required for the binding between these two proteins to take place (32, 46–49). Collectively, our results highlight a new mechanism, whereby TRAF6 acts as a linker between SFKs and cIAP2-dependent IRF-1 K63-linked ubiquitination.

**Figure 6:**
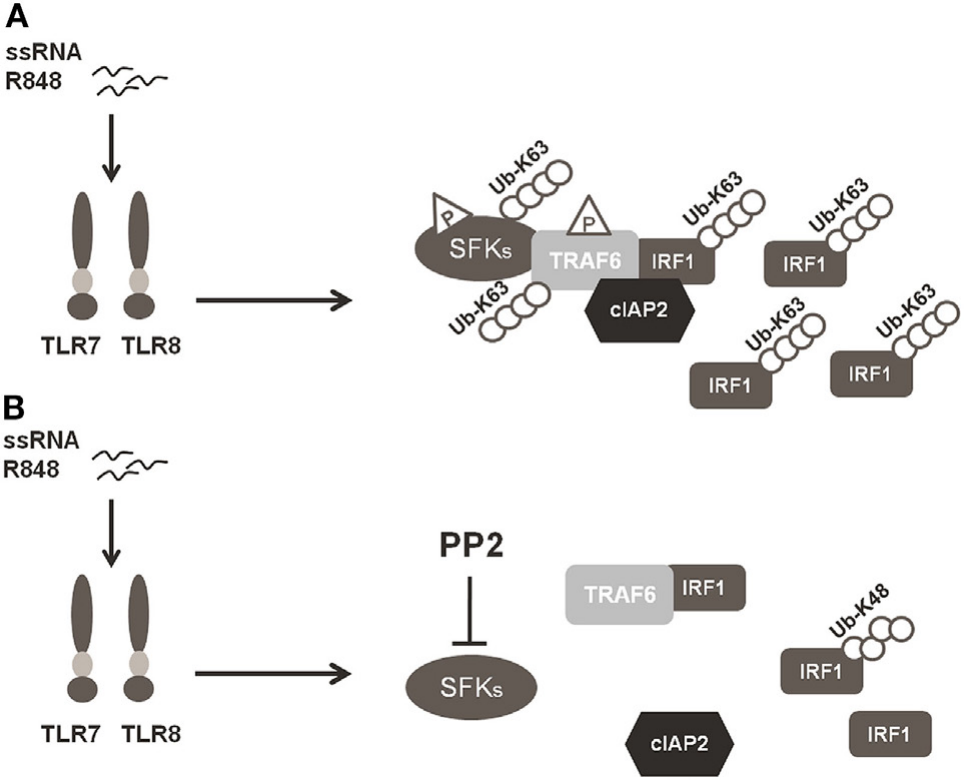
Schematic illustration of SFKs mechanism of action in interferon regulatory factor 1 (IRF-1) K63-Ubiquitination. **(A)** Activation of the TLR7/8 pathway promotes the interaction between SFKs and TNFR-associated factor 6 (TRAF6) allowing the recruitment of both cIAP2 and IRF-1 by TRAF6 with the subsequent K63-ubiquitination and accumulation of IRF-1. **(B)** Preincubation with PP2 impairs the capacity of SFKs to interact with TRAF6 and the formation of the TRAF6-IRF1-cIAP2 complex, inhibiting K63-ubiquitination of IRF-1, which is, however, K48-ubiquitinylated and it is consequently degraded *via* proteasome.

In conclusion, our finding that SFKs are required for K63-linked ubiquitination of IRF-1 and pro-inflammatory cytokine release demonstrates that SFKs activation is a key step in TLR7/8 signaling. In particular, as depicted in Figure 6, the activation of SFKs appears necessary for their own interaction with TRAF6 and for the recruitment of both cIAP2 and IRF-1 by TRAF6. These events lead to the formation of a complex that allows the interaction of cIAP2 and IRF-1 resulting in IRF-1 K63-linked ubiquitination and stabilization.

## Author Contributions

LT, FC, and JV performed and analyzed the experiments; CB and UD designed research studies; LT, FC, CB, and UD wrote the paper. All authors reviewed the results and revised and approved the final version of the manuscript.

## Conflict of Interest Statement

JV is a former employee of the GSK group of companies. UD is an employee of the GSK group of companies and reports ownership of restricted GSK shares. LT was a recipient of a scholarship from GSK Vaccines S.r.l on a grant from MIUR (Medintech training project CTN01_00177_962865). All other authors declare that they have no conflicts of interest with the contents of this article.
