# Supplementary material for: Src Family Kinases Regulate Interferon Regulatory Factor 1 K63 Ubiquitination following Activation by TLR7/8 Vaccine Adjuvant in Human Monocytes and B Cells
Source: Front Immunol. 2018 Mar 1;9:330. doi: 10.3389/fimmu.2018.00330 (PMC5837968; doi:10.3389/fimmu.2018.00330)
Supplement: Supplementary file 1 [file presentation_1.PDF]

*Supplementary Material*

# Src Family Kinases Regulate Interferon Regulatory Factor 1 K63 Ubiquitination following Activation by TLR7/8 Vaccine Adjuvant in Human Monocytes and B Cells

Lorenza Tulli, Francesca Cattaneo, Juliette Vinot, Cosima T. Baldari, Ugo D'Oro*

***Correspondence:**

**Ugo D'Oro**

**ugo.x.doro@gsk.com**

## 1 Supplementary Figures

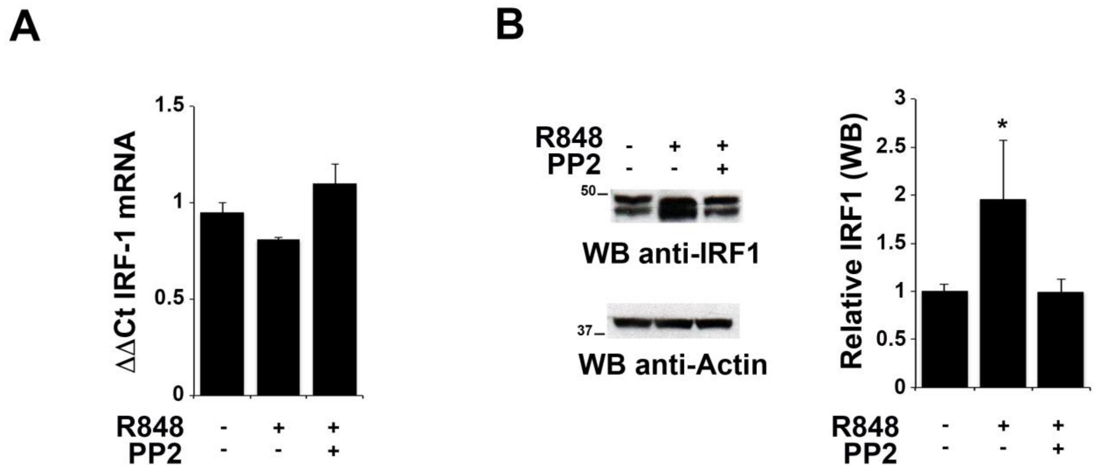


**Supplementary Figure 1. SFKs are required for IRF1 protein accumulation in EBV-B cells.** EBV-B cells were pre-treated or not with PP2 (20 μM) and stimulated with R848 (10 μM) for 2 h **(A)**. qRT-PCR analysis of IRF1 mRNA. The relative abundance of the gene transcripts was determined on triplicate samples from at least 3 independent experiments using the ΔΔCt method and is expressed as the normalized fold expression (mean ±SD) compared to untreated control. **(B)**. Total cell lysates were separated by SDS-PAGE and immunoblotted with anti-IRF1. After stripping, filters were re-probed with an antibody against actin as loading control. Results are representative at least of three independent experiments. *$P<0.05$

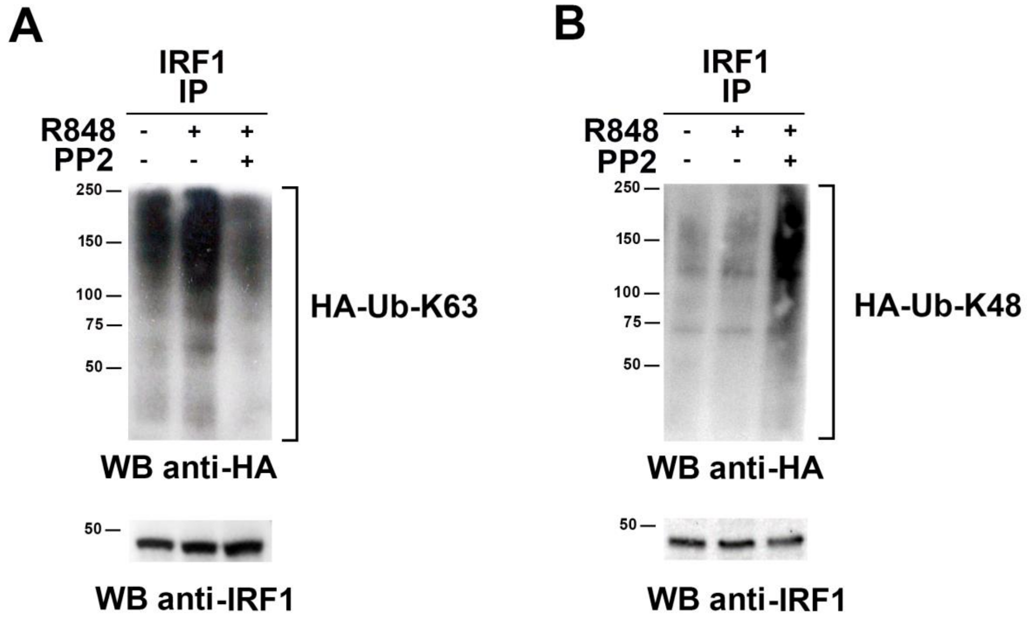


**Supplementary Figure 2. SFKs activation is required for IRF1 K63-linked ubiquitination in THP1.** THP1 cells transiently transfected with plasmids coding for HA-tagged K63-only (A) or K48-only (B) ubiquitin mutants were pre-treated or not with PP2 (20 μM) for 30 min, stimulated for 2 h with R848 (10 μM), then subjected to immunoprecipitation (IP) using an anti-IRF1 antibody and immunoblot using anti- HA antibody for ubiquitin detection. Membrane were stripped and re-probed with anti-IRF1 antibody as control (bottom panels). Results are representative at least of three independent experiments.

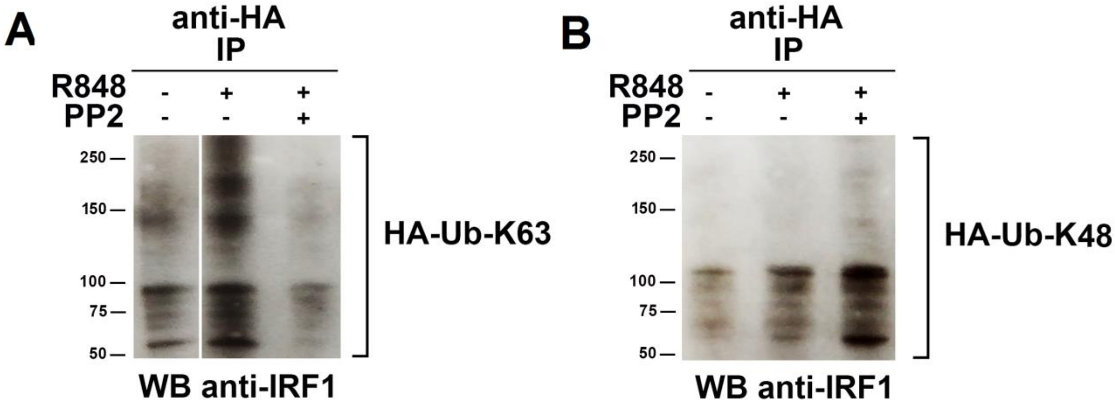


**Supplementary Figure 3. SFKs activation is required for IRF1 K63-linked ubiquitination in hTLR7-HEK293.** hTLR7-HEK293 cells transiently transfected with plasmids coding for HA-tagged K63-only (A) or K48-only (B) ubiquitin mutants were pre-treated or not with PP2 (20 μM) for 30 min, stimulated for 2 h with R848 (10 μM), then subjected to immunoprecipitation (IP) using an anti-HA antibody and immunoblot using anti- IRF-1 antibody for ubiquitin detection. Results are representative of at least of two independent experiments.
